# Prolonged use of dialysis catheters is associated with elevated chronic inflammatory markers: a single center case series

**DOI:** 10.1080/0886022X.2025.2478486

**Published:** 2025-03-17

**Authors:** Mihaly B. Tapolyai, Szabina Czirok, Máté Szász, Ákos Pethő, Tibor Fülöp

**Affiliations:** ^a^ Szent Margit Hospital, Budapest, Hungary; ^b^ Medicine Services, Ralph H. Johnson VAMC, Charleston, South Carolina, USA; ^c^ Department of Internal Medicine and Oncology, Faculty of Medicine, Semmelweis University, Budapest, Hungary

**Keywords:** TDC, dialysis catheters, prolonged catheter use, dialysis access, inflammation

## Abstract

**Background:**

Long-term tunneled-cuffed dialysis catheters (L-TDCs) are typically considered a temporary solution for vascular access in end-stage kidney disease (ESKD). However, a subset of patients relies on L-TDCs for extended periods. This study aims to characterize these patients and evaluate inflammatory markers.

**Methods:**

We conducted a retrospective study, cross-sectional of 13 ESKD patients who had an L-TDC in place for at least 12 months at an urban hospital-based dialysis unit. This group was compared to 26 consecutive patients with stable AVFs. Key parameters analyzed included dialysis efficiency (urea reduction ratio, blood flow rates, venous/arterial pressures) and inflammatory markers (C-reactive protein [CRP]).

**Results:**

L-TDCs remained in use for an average of 40.6 ± 27.5 months (range: upto 113 months). Despite similar dialysis efficiency between groups (urea reduction ratio: 72.5% vs. 68.5%, *p* = 0.07), L-TDC patients exhibited significantly higher CRP levels (19.7 vs. 9.6 mg/L, *p* = 0.03). Dialysis blood flow rates were lower in the L-TDC group (290.3 vs. 339.4 mL/min, *p* < 0.0001), with significantly higher venous (193.5 vs. 149.3 mmHg, *p* = 0.002) and arterial pressures (213.7 vs. 147.5 mmHg, *p* < 0.0001). A moderate correlation between dialysis vintage and CRP was observed in L-TDC users (*r* = 0.383, *p* = 0.17), but not in AVF patients.

**Conclusion:**

Although L-TDC users achieve comparable dialysis efficiency to AVF patients, they experience persistent inflammation, as indicated by significantly elevated CRP levels. These findings underscore the need for closer monitoring and strategies to mitigate long-term inflammatory risks in patients requiring prolonged catheter use.

## Background

Dialysis patients’ vascular accesses have evolved over the last years from various shunts and ports to arterio-venous fistulas (AVF) affording sufficient blood flow through the dialysis machine and delivering an effective dose of dialysis, by removing sufficient waste products form the blood circulated through the blood circuit. Clinical observations and studies have compared effectiveness and safety of the various dialysis accesses and found that long-term use of arterio-venous grafts and dialysis catheters are inferior to AVF, in delivering sufficient dose of dialysis. Furthermore, the use of artificial implanted materials are plagued with an increased risk of access-related blood stream infections, with the highest risk occurring in those with temporary or tunneled-cuffed dialysis catheter (TDC) as well as there are many more instances of access related blood stream infections with the implanted devices, may that be a dialysis graft or a tunneled dialysis catheter (TDC). The Dialysis Outcomes and Practice Patterns Study (DOPPS), a clinical observational study [1] even found that the relative risk (RR) of death in dialysis units using TDC’s in more than 28% of their patients compared to units with less than 7% of usage was 1.28 with a statistically significant p value of 0.01. For these reasons, the 2006 update of the DOQI (National Kidney Foundation Kidney Disease Outcomes Quality Initiative) Guidelines [2] recommend that ‘Patients with CKD stage 5 should be educated on the risks and benefits associated with catheters and strongly encouraged to allow the evaluation for and creation of a fistula for long-term access when appropriate’ [2].

Notwithstanding the well-meaning recommendations, there are still a number of patients whose sole dialysis access remains a catheter. Often, these patients needed an immediate start of renal replacement therapy and they will thus be dialyzed through a catheter; other subjects were not candidates for an AVF due to arm anatomy, failure to mature AVF properly or experienced excessive AVF enlargement with high-output heart failure developing. In some areas, the shortage of skilled surgeons creating such fistulas is an additional concern. Some patients may even upright refuse AV access placement, to displace a functioning TDC. Ultimately, many patients end up having a TDC for permanent access. While the hope is that these patients eventually will get an AVF, but there is a select few patients who have a long-term use of their TDC. There is, however, a small population of patients who will be chronically dialyzed *via* a TDC despite professional recommendations. While unusual, some patients can have their TDC for more than one year in place. We report the case of 13 such patients with long-term TDC use (L-TDC) and compare them to 26 of their fellow dialysis patients with a working AVF in the same dialysis unit. We attempted to characterize these patients and see if there were any untoward consequences of L-TDC.

## Methods

This is a retrospective, case-control, cross sectional study based on existing data without an intervention or treatment change for the sake of the study. The study was approved by the Hungarian Medical Research Council for ethical consideration and approval (TUKEB # BM/27893/2024) was obtained. Patients’ laboratory values were obtained from their monthly, regularly scheduled blood works and their dialysis and demographic data as well as the information regarding their dialysis catheters were acquired from patient records. Patients’ privacy was maximally guarded, their data was used only in cumulative results. Inclusion criteria were either the presence of a fully functional and well- working AVF defined as a fistula that could be cannulated with 2 needles and had achieved at least a 300 mL/min blood flow with a venous pressure of less than 60% of the blood flow, or a L-TDC they had to have this catheter for at least 12 months. The comparator patients with the well working AVF were treated in the same dialysis unit during the same shift as those with the L-TDC. The catheters were not exchanged during the preceding time-period, they had the originally placed catheter. Exclusion criteria were defined as those patients who had a temporary catheter or their TDC had been in place for less than a year. The patients’ monthly laboratory tests were used for the study drawn the same month time for all. For statistical methods we used two tailed unpaired Student’s t test as well as univariate linear regression analysis of Microsoft Excel (Microsoft Professional Plus 2016). Statistical significance was defined as a ‘p’ value of less than 0.05.

## Results

There were 13 patients in the L-TDC group whom we compared with 26 AVF patients. All patients with a L-TDC had two single-lumen Tesio catheters (Medcomp; Harleysville, PA, USA) made of soft radiopaque polyurethane. Notwithstanding we did not exclude any patients from analysis with L-TDC but we stopped collecting data of AVF patients once we reached the 1:2 ratio for L-TDC: AVF patient numbers. There were 9 (69.2%) left sided catheters inserted to the left internal jugular vein and there were 4 (30.8%) patients whose L-TDC was inserted on the right side. Eight (61.5%) of the 13 catheter tips were confirmed to be in the right atrium by chest X-ray, 3 (23.0%) at the superior vena cava – right atrium junction and 2 (15.4%) were in the superior vena cava.

The demographic characteristics (Table 1) of the L-TDC group and the AVF group was very similar, understandably so since the same nephrologists treated them at the same teaching-hospital based dialysis unit. They were comparable in age, gender, dialysis vintage (years), serum albumin, hematocrit and erythropoietin (EPO) monthly dose. As expected, there was a very significant difference between the dialysis machine pump speeds and the transducer pressures, venous and arterial as well. Contrary to our expectations patients in the L-TDC group had a non-inferior quality of dialysis as measured by the Urea Reduction Rate (URR), in fact the L-TDC patients had a noticeably higher URR though statistically not reaching significance (p: 0.07). According to the Kolmogorov-Smirnov test of normality, CRP data for the L-TDC cohort are normally distributed: skewness: 1.087; kurtosis −0.241; the value of the K-S test statistic (D) is 0.24497. Similarly, the data are normally distributed for the AVF group as well (D: 0.22202; skewness: 2.156556 and kurtosis: 5.751626. L-TDC patients achieved a better hematocrit: 35.7% vs 32.0% p: 0.03 – while receiving essentially the same amount of monthly EPO dose (16,153.0 Units vs. 16,640.0 Units p: ns).

On the negative side, L-TDC patients have a progressively worse C-reactive protein (CRP) increasing by the number of dialysis vintage years, with; a coefficient of correlation (r) for the L-TDC group between dialysis vintage (years) and CRP 0.383 (*p* = 0.17); whereas there was no such correlation seen in the AVF group. Another marker of inflammation, the neutrophil/lymphocyte ratio (5.0 vs 4.0) was also higher in the L-TDC group, however, this did not reach statistical significance. Finally, the cardiovascular risk indicator of BUN/albumin ratios did not show a significant or appreciable difference.

No patients with L-TDC were excluded from this analysis.

**Table 1.** Demographics and results.

	L-TDC (n: 13)	AVF (n: 26)	p
Age, years (median)	71.7 (74)	64.9 (68)	ns
Sex (% men)	38.4	57.7	ns
Dialysis vintage, years (median)	5.3 (3.5)	4.0 (4.0)	ns
Dialysis blood flow (Qb) (median)	290.3 (280)	339.4 (340)	<0.0001
Venous transducer pressure, mmHg (median)	193.5 (196)	149.3 (155.5)	0.0002
Arterial transducer pressure, mmHg (median)	213.7 (220)	147.5 (149.5)	0.0001
Urea reduction ratio (median)	72.5 (74,3)	68.5 (67.9)	0.070
Albumin (mg/dL) (median)	3.8 (3.9)	3.9 (3.9)	ns
BUN/Albumin (median)	5.8 (6.1)	5.6 (5.3)	ns
Hematocrit (%) (median)	35.7 (35.2)	32.0 (32.1)	0.036
EPO dose (units/month) (median)	16,153.0 (20000)	16,640.0 (20000)	ns
neutrophils % (median)	71.8 (73.8)	66.4 (64.8)	0.057
lymphocyte % (median)	16.1 (14.3)	19.8 (18.5)	ns
neutrophil/lymphocyte (median)	5.0 (5.2)	4.0 (3.5)	ns
CRP (mg/L) (median)	19.7 (13.3)	9.6 (5.0)	0.039

## Discussion

Dialysis catheters are the least preferred access types for many reasons. Perhaps the greatest reason is that dialysis units who have a larger proportions of patients with dialysis catheters will have a higher rate of mortality, as shown in the Dialysis Outcomes and Practice Patterns Study (DOPPS) [1] that found a higher rate of patients dying with such access. Notwithstanding, there are studies showing that even temporary dialysis catheters can function acceptably as described by other authors, including a similar cohort of patients in a Croatian cohort. [3]. They found that these patients in Croatia with catheters had a low infection rate and a good functional long-term vascular access. Another observational study [4] at the Boston Medical Center found that out of 600 TDC placement 50 patients had their catheter for longer than 180 days and that patient group had ‘no significant difference in survival at 24 months for the long-term TDC group compared with the comparator group (93.6% vs 92.7%; *p* = 0.28)’. Their infection rate was not any higher that of those who had their catheters for shorter time, an observation also reported in the paper referenced above with temporary catheters.

While the National Kidney Foundation Vascular Access Work Group [5] recommends catheter exchanged when dialysis catheters become dysfunctional or poorly functional, we had no such dysfunctional catheter and none of our patients experienced catheter related infections. We can confirm that the dialysis catheter functionality was preserved for up to 113 months. Our patients did achieve an equal or URR than those with an AVF, though it did not reach statistical significance (72.5 ± 5.9% [95% CI: 69.2– 75.7] vs. 68.5 ± 6.3% p: 0.070 [95% CI: 66.1–71.0]). This perhaps was due to the practice of our dialysis nurses to ‘start dialysis slow’ for those with an AVF and using size 16 gauge needles in about 50% of patients with an AVF. Our L-TDC patients did not require more erythropoietin (16,153 ± 1066 units/month vs 16,640 ± 9638 units/month p: 0.88) with better hematocrits (p: 0.03) in the L-TDC group. Their dialysis pump speeds were decidedly lower in the L-TDC group and the transducer pressures much higher to a very high degree of statistical significance, however, without apparent clinical consequences. These L-TDC patients had no catheter related complications the preceding periods.

There was no negative consequence of L-TDC’s when serum albumins were investigated (5.8 ± 0.35 vs. 5.6 ± 0.23 mg/dL p: ns) in favor of the L-TDC though both median values being exactly 3.9 mg/dL. The BUN/albumin ratios, which are a known index for cardiovascular events [6–8] in various clinical settings were similarly indistinguishable at 5.8 ± 1.8 vs. 5.6 ± 1.6 p: ns. However, we found that the CRP were significantly higher in the L-TDC group compared to the AV cohort; mean: 19.8 ± 18.7 [CI: 9.6–29.9] vs. 9.6 ± 11.0 [CI: 5.383–13.885] p:0.03 with a wide separation of medians: 13.3 vs. 5.0. Not only did this indicator of inflammation distinguish the two cohorts from one another in aggregate data, but there appeared to be a trend with time; there was an increase of CRP with the increase of exposure to catheter with respect of dialysis vintage (years). The linear regression coefficient of correlation for the L-TDC group between dialysis vintage and CRP was 0.383; there was no such correlation for the AVF group (p: ns). A chronic progressively worsening inflammation can be gleaned from these results, however, without a clear understanding of its significance as the previously mentioned clinical studies with much larger patient cohorts found no survival differences.

We recognize the many limitations of our paper, including the single-center design, limited number of enrollees and obvious survival bias of observed L-TDC. Care may not be uniform across all units – e.g., Hungarian units are utilizing exclusively registered nurses for dialysis and access care and the concept of dialysis patient care technician is unknown. Thus, while recognizing the many limitation of dialysis catheters, our observation transmit valuable observation highly applicable to the local environment. We think the use of L-TDC is neither a recommended nor a good access type because of chronic inflammation and other risks discussed above, but this access can work well in select patients without the possibility for an AV Fistula.
